# 3D-Printed Polycaprolactone Mechanical Characterization and Suitability Assessment for Producing Wrist–Hand Orthoses

**DOI:** 10.3390/polym15030576

**Published:** 2023-01-22

**Authors:** Diana Popescu, Constantin Stochioiu, Florin Baciu, Mariana Cristiana Iacob

**Affiliations:** 1 Department of Robotics and Production Systems, Faculty of Industrial Engineering and Robotics, University Politehnica of Bucharest, 060042 Bucharest, Romania; 2 Department of Strength of Materials, Faculty of Industrial Engineering and Robotics, University Politehnica of Bucharest, 060042 Bucharest, Romania

**Keywords:** polycaprolactone, 3D printing, mechanical properties, digital imaging correlation, UV-B exposure, wrist–hand orthosis

## Abstract

In this research, the mechanical properties of 3D-printed polycaprolactone (PCL), a biocompatible and biodegradable semi-crystalline polyester, available as feedstock for additive manufacturing technology based on the material extrusion process, were determined. The influence of the infill pattern (zig-zag vs. gyroid) and ultraviolet (UV-B) exposure over the specimens’ mechanical performances were also investigated to gather relevant data on the process parameter settings for different applications. Specimens and samples of 3D-printed PCL were analyzed through tensile and flexural tests. The experimental data showed the good repeatability of the manufacturing process, as well as a mechanical behavior independent of the specimens’ infill pattern at full density. No differences between the failure patterns of the tensile specimens were recorded. UV-B exposure proved to have a significant negative impact on the specimens’ tensile strength. The 3D printing of PCL and PCL blends is reported mainly for use in scaffold manufacturing or drug delivery applications. As another novelty, the suitability of commercial PCL filaments for producing patient-customized wrist–hand orthoses was also assessed in this study. Semi-cylindrical PCL samples mimicking the forearm part of a wrist–hand orthosis with hexagonal open pockets were 3D-printed and mechanically tested. The results were discussed in comparison to samples with a similar design, made of polylactic acid. The experiments revealed the need to carefully calibrate the manufacturing parameters to generate defect-free, good quality prints. Once settings were established, promising results were obtained when producing orthoses in a ready-to-use form. On the other hand, the attempts to thermoform flat 3D-printed PCL orthoses proved unsuccessful.

## 1. Introduction

Poly(ε-caprolactone) (PCL) is an environmentally friendly polyester, semi-crystalline at room or body temperature, attracting researchers’ attention due to its biocompatibility, biodegradability, and reduced immunogenicity [1]. PCL glass transition temperature is around −60 °C, whereas the melting temperature is around 60 °C [2]. The use of PCL or PCL blends as feedstock in additive manufacturing technology based on the material extrusion process (MEAM, also referred to as 3D Printing—3DP) is reported mainly for applications related to drug delivery or scaffold manufacturing [3,4]. In the existing studies, 3D prints with properties suitable for the bone tissue engineering field are fabricated from specially prepared materials based on PCL [5,6,7,8,9]. An interesting study using state-of-the-art analysis on PCL mixed with antimicrobial metals for 3D-printed customized wound dressing applications [10] was also found. However, there are no reports on the use of 3D-printed PCL for orthotics applications, although the antimicrobial properties can be useful for patients with skin problems or burns, for instance. Actually, in the literature, there is not much information on how to create defect-free PCL parts at a tenth of mm scale, or on the influence of process parameter settings on the mechanical performances of PCL specimens or prints [11]. So far, no paper has addressed the full mechanical characterization of the 3D-printed specimens made of commercially available PCL filaments in different conditions related to the manufacturing parameters or UV-B exposure, although such knowledge is relevant when designing and producing prints for diverse applications. In this context, the current research aimed firstly at determining the mechanical characteristics of 3D-printed PCL specimens (using Young’s modulus, Poisson’s coefficient, apparent yield stress, ultimate tensile stress, and characteristic curves). The effect of the infill pattern (gyroid vs. zig-zag) and UV-B aging treatment on the mechanical properties were also investigated. Then, semi-cylindrical PCL samples with hexagonal open pockets mimicking the forearm part of a wrist–hand orthosis (WHO) [12,13] were 3D-printed in a vertical position and subjected to flexural tests [14]. The experimental results were discussed in comparison to polylactic acid (PLA) samples with a similar design, with PLA being the most common material for the 3D printing of upper limb orthoses and prostheses [15]. Numerical simulations were also conducted as they are considered useful for designers in establishing the orthosis designs (thickness, pockets). The finite elements-based model was validated based on the gathered experimental data. 

All these investigations represent novelties in the field of 3D printing of commercial PCL polymers, with the material characterization from many perspectives (mechanical, manufacturing parameter influence, deformability, UV-B aging) being supplemented with a practical application in the orthotic field.

WHOs are frequently recommended medical devices because of the high occurrence of wrist-related injuries [16]. Depending on the diagnosis (arthritis, fracture, strains, etc. [17]), splints, braces, or casts [18] are used for different periods of time, and functionality and comfort (to enhance compliance to wear the WHO) are the main criteria used in their development [19,20]. An attractive design, customization based on the patient’s forearm anatomy (usually acquired by 3D scanning [21,22]), and a lightweight design in comparison to traditional casts or splints are the frequently mentioned advantages of 3D-printed WHOs (3DP-WHOs) [23,24]. Usually these orthoses are built vertically (in their ready-to-use form) using the MEAM process, but there is also the alternative of producing WHOs in a flat 3D-printed form and thermoforming them in a warm environment for the subsequent form-fit on the patient forearm [25,26]. The rationale behind studying the use of PCL filaments for such an application is related to the low melting temperature of this material, which makes embedding sensors possible (to monitor when the WHO is worn [27] or therapeutic magnets that might alleviate pain [28]), acknowledging that these devices are temperature sensitive. Moreover, the low softening temperature of PCL could be a benefit in the case of thermoformable 3DP WHOs because no special care will be required to manipulate the flat orthosis and form it directly on the patient skin. PCL also has antimicrobial properties which represent another reason for our investigations, as already mentioned. Additionally, PCL is hydrophobic, with this characteristic sustaining the daily usage of WHOs which involves the contact of the orthosis with water (for hygiene purposes, for example).

## 2. Materials and Methods

The following steps describe the methodology applied in this research:

- Identify the optimal settings (bed and printing temperatures, printing speed) to assure that the test specimens and the samples do not have defects (voids between the deposited threads, over extrusion, delamination, or warpage);
- Use 3D printing to produce the specimens and other test samples;
- Expose the tensile samples to UV-B irradiation;
- Subject the 3D-printed PCL specimens to tensile testing (digital imaging correlation investigations) and flexural testing to determine their mechanical characteristics;
- Use 3D printing to produce the semi-cylindrical samples made of PCL and PLA of different thicknesses, and then subject them to bending tests;
Develop and validate a finite elements-based model for the semi-cylindrical part of the WHOs;
- Gather and discuss the results.

### 2.1. Manufacturing 3D Prints for Tests

As mentioned, the literature lacks relevant data on the process parameter settings for 3D prints made of PCL filaments, and their relationship with different characteristics such as mechanical behavior, surface quality, dimensional accuracy, or aging. Three-dimensional-printed patient-customized nose and ear wound dressings, 1 mm thick, designed initially using 3D scanning data, were investigated by Muwaffak et al. [10]. To fulfill their functional purpose, these prints were made of blends of PCL and antimicrobial metals such as zinc, silver, and copper. The 3DP parameters set in the study were the following: 0.1 mm layer thickness, 170 °C extrusion temperature, 100% infill, 2 shells, and 50 mm/s printing speed. A recent study [29] focused on comparatively analyzing PCL and PCL with natural additives, and characterizing, from thermal and mechanical standpoints, the 3D-printed specimens made of these materials. The PCL specimens were 3D printed at an 80 °C nozzle temperature and a 40 °C bed temperature. No other information on the printing parameters was provided. Mehraein [11] conducted experiments to find the optimal parameter settings to obtain good quality PCL prints. The selected combination of parameters in [11] was: 90% infill density, 0.1 mm layer thickness, 2 shells, 21 °C bed temperature, 165 °C nozzle temperature, 55 mm/s printing speed, and 2.97 mm^3^/s flow rate. The author also determined the specimens’ ultimate tensile strengths and stiffnesses. Different bed temperatures and printing speeds were investigated in [11], observing that these do not influence the quality of the prints (visually assessed). Regarding the parameter settings, an important aspect noted in Mehraein’s study was that these were quite different from their filament producer recommendation (3D4Maker, Sweden). Under these conditions, and as we used PCL filaments from a different producer than in [11], it was necessary to start our investigations with a thorough calibration/optimization of the printing parameter settings. 

**Figure 1:**
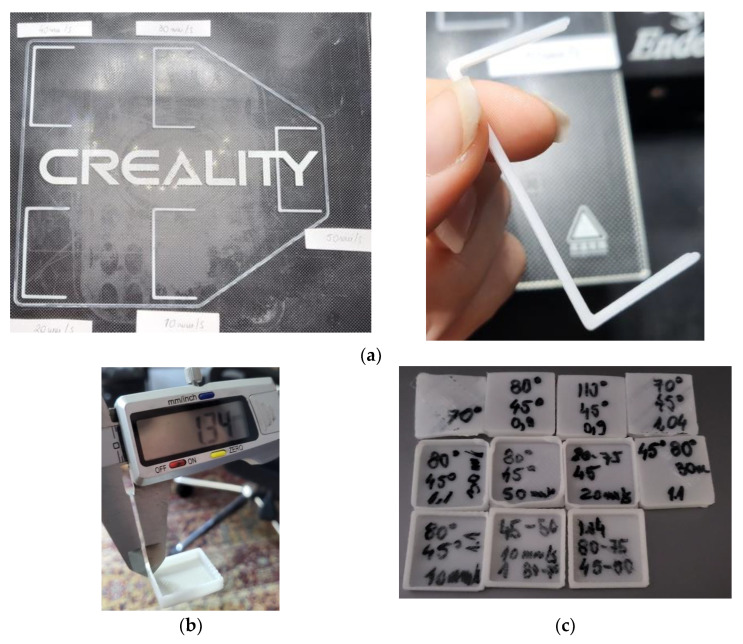
Representative images with the samples used for the parameters’ calibration: C-lines (**a**); calibration cube measurement (**b**); calibration cubes for different parameter settings (**c**).

For calibration, C-lines (50 mm × 25 mm) and 10 mm long cubes were used as in [30] (Figure 1). Table 1 presents the tested values for the printing speed, bed and extrusion temperatures, and flow rate. The room temperature was 23 °C.

All prints were manufactured in a Creality Ender 3 Pro 3D printer (Shenzhen Creality 3D Technology, Shenzhen, China) using Cura Ultimaker (Ultimaker BV, Utrecht, The Netherlands) as the slicing software. The filaments were natural eMate PCL (ESun, Shenzhen, China), with a 1.75 mm diameter, 1.16 g/cm³ density, and 60 °C recommended temperature for reshaping with water. In order to 3DP below 170 °C, the 3D printer’s command software had to be adapted to allow cold extrusion. 

The following process parameters were kept constant during the print manufacturing: layer thickness: 0.1 mm; infill density: 100%; shells: 2; and tip/bottom layers: 2. The C-lines (Figure 1a) and the calibration cubes (Figure 1b,c) were measured and their quality was visually assessed. The following parameter settings ensured the best results: bed temperature of 45 °C for the first layer and 50 °C for the other layers; printing temperature of 80 °C for the first layer and 75 °C for the other layers; printing speed of 20 mm/s.

**Figure 2:**
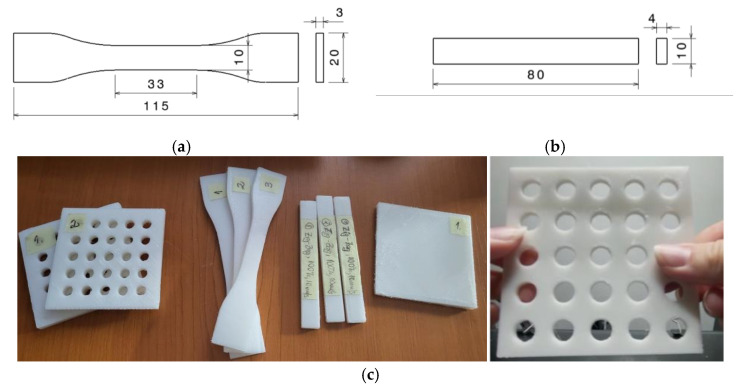
Tensile specimen dimensions (**a**), flexural specimen dimensions (**b**), examples of prints for tests: tensile and flexural specimens, different PCL samples (open pockets samples, tensile samples, bending samples, cuboid samples) (**c**).

Tensile (Figure 2a) and flexural specimens (Figure 2b) with gyroid and zig-zag infill patterns at full infill density were manufactured using the above mentioned settings. 

Three zig-zag specimens were exposed to UV-B for 9 h before the mechanical tests. For use in the testing of the thermoforming process, different samples with a rectangular shape (60 mm × 60 mm × 5 mm; 90 mm × 90 mm × 3 mm; 90 mm × 90 mm × 2 mm; 90 mm × 90 mm × 1.8 mm), with or without open pockets, were also manufactured using the same printing parameters. Figure 2 presents some of these specimens and samples (Figure 2c), as well as the dimensions of the tensile and flexural specimens (Figure 2a,b).

**Figure 3:**
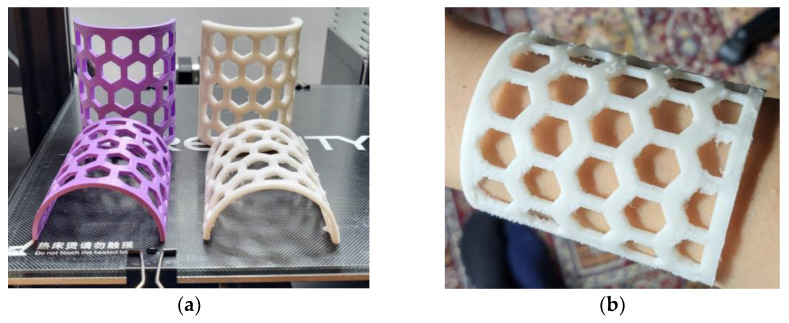
Three-dimensional-printed PCL (white) and PLA (purple) semi-cylindrical samples (**a**); PCL sample mimicking the arm part of a 3DP WHO placed on a user’s forearm (**b**).

Additionally, semi-cylindrical samples with a 50 mm diameter, 70 mm height, and different thicknesses (2 mm, 3mm, and 4 mm), as well as 6 mm hexagonal open pockets, were 3D-printed in a vertical position, using PCL and PLA (Devil Design, Mikołów, Poland) (Figure 3). Their design mimicked the typical design of 3DP WHOs in the forearm section.

### 2.2. UV-B Radiation Exposure

Three tensile samples with a zig-zag infill pattern were exposed to UV-B for 9 h using UV-MAT equipment (Opsytec, Ettlingen, Germany), with an irradiance of 5 mW/cm². The PCL specimens’ main dimensions were measured before and after the irradiation to evaluate the effect of UV-B aging treatment over their dimensional accuracy.

### 2.3. Mechanical Testing

**Figure 4:**
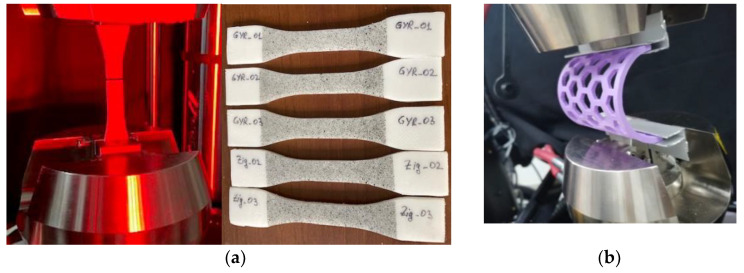
Mechanical test set-ups: DIC tensile (**a**); bending of the semi-cylindrical samples (**b**).

The experimental set-ups and the specimens prepared for the mechanical tests performed in this research are presented in Figure 4. Nine tensile specimens (three with a gyroid infill pattern, and six with a zig-zag infill pattern from which three were UV-B irradiated) were subjected to uniaxial loading up to failure to determine the stress–strain curve, Young’s modulus, Poisson’s ratio, apparent yield stress at 0.2% permanent strain, and ultimate tensile strength. Loading was achieved with an Instron 8872 electro-hydraulic Universal Testing Machine with a load speed of 1 mm/min, while strain recording was achieved with the digital image correlation (DIC) technique using the Dantec Dynamics DIC system (Figure 4a). Prior to testing, the PCL specimens were coated with a layer of white paint, followed by a black speckle for the DIC technique.

The PCL specimens with zig-zag and gyroid infill patterns were analyzed by means of their mechanical behavior under 3-point bending tests. To that end, three samples for each batch were subjected to the respective test, with a loading speed of 4 mm/min on the same Instron equipment. The distance between the supports was 60 mm.

Stress (*σ*) was computed as the maximum normal stress under the calculated bending moment (Equation (1)), and strain (*ε*) as the maximum strain in the section with the maximum deflection (Equation (2)), according to the ASTM D790 standard [31], based on the classical beam theory applied to three-point bending test:

(1) $$\sigma =\frac{3PL}{2bd^{2}}$$

(2) $$\varepsilon =\frac{6Dd}{L^{2}}$$

where *P* is the recorded force representing the load on the force-displacement curve at a given point, L is the support span distance, *b* is the sample width of the specimen, *d* is the specimen’s thickness, and *D* is the deflection in the mid-span.

The overall rigidity of the semi-cylindrical orthoses was compared (PLA samples of 2 mm and 3 mm thickness vs. PCL samples of 2 mm and 4 mm thickness) after subjecting them to the bending tests (Figure 4b). The purpose was to investigate if the 3D-printed PLA wrist–hand orthoses could be replaced with PCL orthoses in terms of provided stiffness, and in which conditions (meaning orthoses’ thickness values) this replacement could be made.

### 2.4. Model Simulations with Finite Elements Method

The digital version of the semi-cylindrical sample, designed to be 3D printed, was loaded into ANSYS (ANSYS Inc., Canonsburg, USA) to perform finite elements-based simulations to validate the model. For validation, two 3D-printed PCL models with thicknesses of 2 mm and 4 mm were used. 

**Figure 5:**
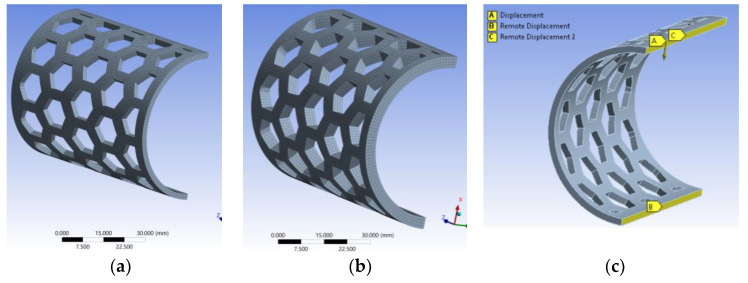
Meshing the models: 2 mm thick model (**a**); 4 mm thick model (**b**); external displacement and constraints (**c**).

The models were meshed using the SOLID 186 type element with 20 nodes with three degrees of freedom each. After the meshing process, the models were divided into a network of elements consisting of 69,273 nodes and 12,333 elements for the model with a 2 mm thickness (Figure 5a), and 90,805 nodes and 17,076 elements for the one with a 4 mm thickness (Figure 5b).

For each semi-orthosis model, the displacement obtained as a result of the experiments was imposed (displacement-controlled test), and the force required to obtain such displacement was recorded. The bonding conditions in the models were set according to the clamping system in the test equipment and samples positions (Figure 5c).

## 3. Results

### 3.1. PCL Printability

During the initial tests focused on the 3DP parameter optimization for quality (form, dimension, visual aspect, defect-free), several aspects were observed. There was no significant difference in terms of dimensions, deposition quality, and uniformity of the deposited filament width between the C-lines 3D printed with 10 mm/s, 20 mm/s and 30 mm/s at a flow of 1 mm^3^/s. This confirmed Mehraein’s [11] observation discussed in Section 2.1. An increase in the printing speed should be correlated with an increase in the flow rate to obtain conformal lines. Again, this was in agreement with Mehraein’s optimal settings (55 mm/s printing speed and 2.97 mm^3^/s flow rate) [11]. However, in our study, when 3D printing the calibration cubes, when all the specimens and samples had more than a 4 mm height, only the 20 mm/s printing speed produced good quality specimens. 

**Figure 6:**
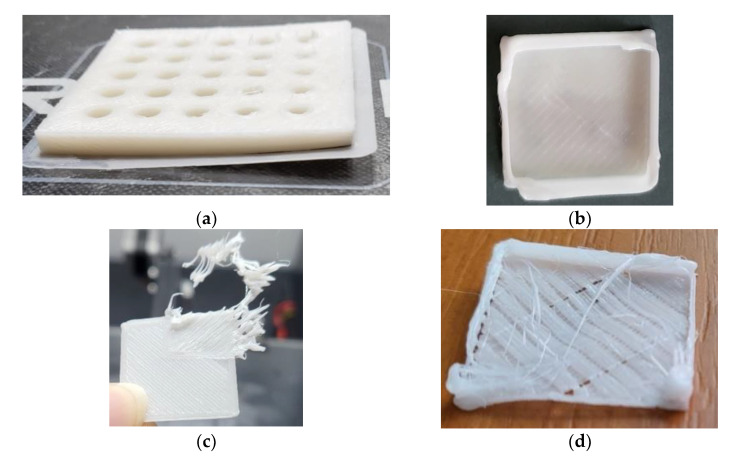
Optimization of 3DP process parameters. Defects: warpage (**a**); over-extrusion (**b**); sample adherence to the nozzle (**c**); under-extrusion, and sample adherence to the nozzle (**d**).

Another observation was that higher printing speeds had a negative influence over the quality of the 3D prints’ corners. Moreover, the optimal bed temperature identified in the C-line tests did not prove optimal for the thicker samples as well, as they had the tendency to contract and detach from the platform (warpage, Figure 6a), or to not cool fast enough, thus causing the threads to collapse (Figure 6b). Using a non-heated bed, as suggested by the PCL filament producer, was not a suitable option in our study because the 3D printer had a glass bed which also contributed to the first layer adhesion problems. As a matter of fact, the adhesion problems were encountered even when using the heated bed, with paper stick glue being needed in the initial tests. Thus, there was the issue of warpage on the one hand, and then there was the issue of the PCL sticking to the printing nozzle as a result of the too-slow cooling, leading to different flaws (Figure 6c,d). In this context, the decision to disable cooling for the first four layers and then automatically increase it to 100% was made based on the experience gathered in the calibration phase. This resolved the problems and provided good quality 3D-printed specimens as can be seen in Figure 2.

In the Muwaffak et al. [10] and Mehraein [11] studies, the extrusion temperature was set to 170 °C and 165 °C, respectively. In our research, the maximal value set for the printing temperature was 110 °C because our interest was to receive the benefit of the low melting temperature as explained in the introduction. Moreover, as first the layer adhesion problems and over-melting were observed at 110 °C and 50 mm/s, the decision was made not to test a higher printing temperature. 

It is also worth mentioning that the quality of the 2 mm thick PCL semi-cylindrical samples was not as good as the quality of 4 mm thick PCL semi-cylindrical samples (Figure 3). For the 2 mm thick samples, bridging (i.e., horizontally passing between two disconnected zones/points) problems occurred as a result of the insufficient cooling of the deposited PCL threads.

### 3.2. PCL Sample Thermoforming

**Figure 7:**
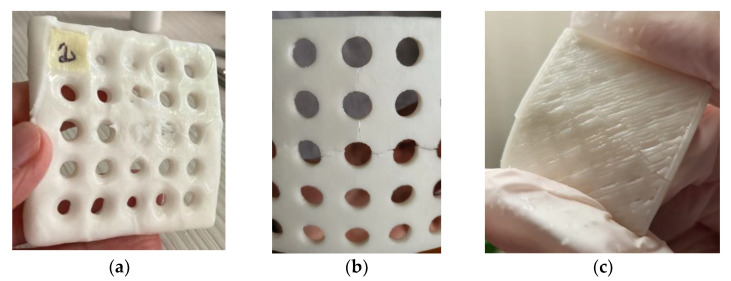
Three-dimensional-printed PCL samples. Thermoforming results: melted sample (**a**); open pocket sample with fractures caused by the deformation attempts (**b**); lower infill density sample with fracture line caused by deformation (**c**).

Several 3D-printed samples of different thicknesses (1.8 mm, 2 mm, 3 mm, and 5 mm) were tested for thermoforming. Based on the PCL filament producer’s data, this material can be reshaped in warm water at 60 °C. However, all the attempts to remodel the samples after immersion in water at different temperatures (50 °C, 55 °C, 60 °C, 65 °C, and 70 °C) did not produce the desired results. Figure 7a presents an example of a 5 mm thick sample that melted and became sticky after immersion in 60 °C water, while other thinner samples with different infill densities (Figure 7b,c), which were warmed at 50 °C and 55 °C, cracked during their molding around a cylindrical shape. It was observed that at temperatures higher than 60 °C, the samples’ surfaces melt, with a barrier against further softening of the interior being created. Thus, the deformation process produced flaws.

### 3.3. PCL 3D Prints’ Visual and Dimensional Changes after UV-B Exposure

Table 2 presents the dimensions of the zig-zag tensile specimens before and after the UV-B radiation, proving that the 3D-printed PCL are dimensionally stable under irradiation up to 9 h. No similar investigations were identified in the literature to compare these findings with. Further work is needed to document the effect of UV-B on the dimensional stability for longer aging periods.

**Figure 8:**
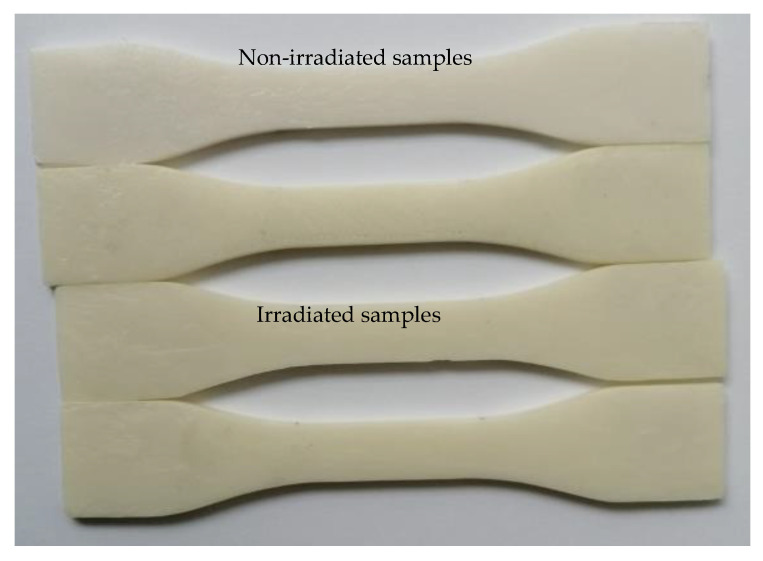
Light yellowing of the PCL samples after 9 h of UV-B irradiation in comparison to a white, non-irradiated sample.

In [32], injected molded PCL samples were subjected to UV-B exposure for up to nine weeks and then characterized, with photodegradation being observed. In our case, even for a much shorter period of exposure, a slight change in the color of the specimens was visible as well (Figure 8). No degradation of the surface (cracks, increase in the roughness) was noticed. As mentioned in Franca et al. [32], the photodegradation mechanism can be attributed to the presence in the PCL polymer chain of chromophores that absorb radiation and start free radical oxidation, and then chain scission reactions [33,34]. 

### 3.4. Mechanical Tests Results

**Figure 9:**
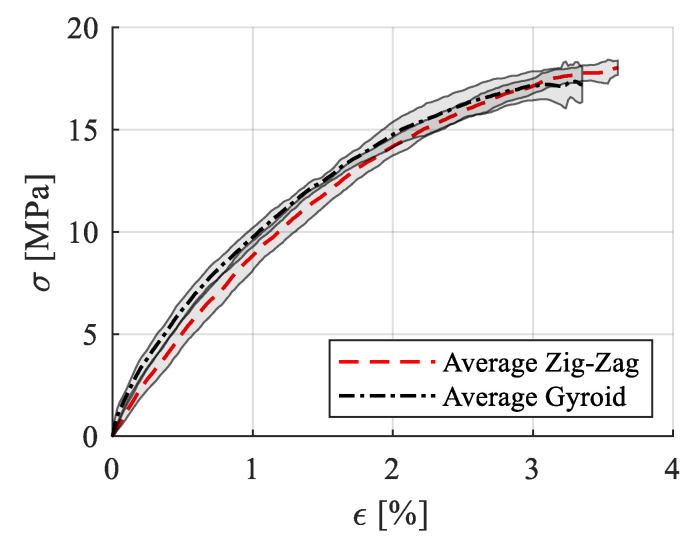
Stress–strain variation curves for the tensile specimens (zig-zag, gyroid, zig-zag after UV-B exposure).

In Figure 9, the average stress–strain curve with standard deviation is presented, while in Table 3, the calculated mechanical properties as means and standard deviation are listed (0.5–1% strain range).

**Figure 10:**
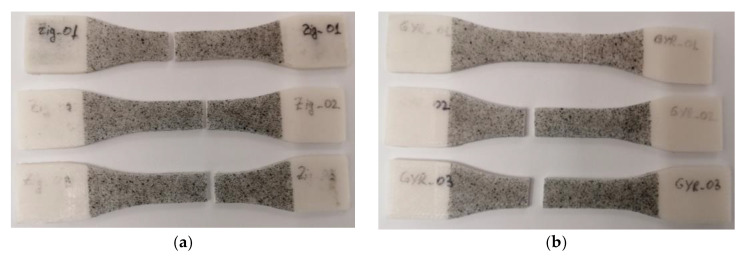
Failure patterns of the 3D-printed PCL: zig-zag tensile specimens (**a**); gyroid tensile specimens (**b**).

The experimental results showed that the infill pattern at full density (100% for the zig-zag model and 99% for the gyroid model as set in the Cura slicer) had no significant impact on the mechanical properties or on the fracture pattern. Moreover, the 3D-printed PCL specimens were proven to have an excellent repeatability in the manufacturing process, both in terms of mechanical properties and dimensional accuracy (Table 2 and Table 3). All the tensile samples broke within the gauge area with a straight pattern (Figure 10), regardless of the infill model, and without much plastic deformation (necking), which indicated a brittle fracture. The UTS (ultimate tensile strength) value of PCL specimens was around 18 MPa, which confirmed the filament producer’s data [35], as well as the results of Mehraein (16.086 MPa at 100% infill density) [11].

**Figure 11:**
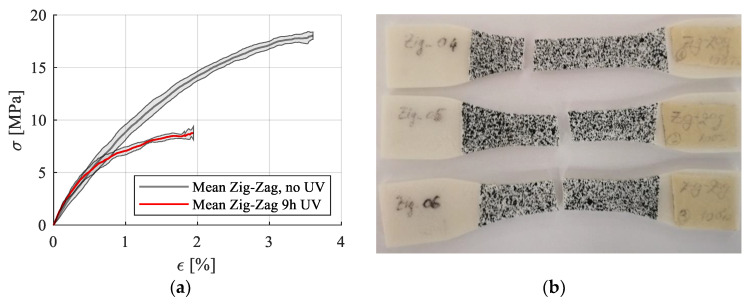
Stress–strain curves for zig-zag printing pattern with no UVB treatment and with UV treatment (**a**); Failure patterns for the UV-B treated samples (**b**).

UV-B exposure for 9 h negatively influenced the mechanical behavior of the analyzed zig-zag specimens. The average stress–strain curve with standard deviation is presented in Figure 11, and the calculated mechanical properties are presented in Table 4. For these specimens, the mechanical properties have been calculated in the 0.15–0.3% strain domain due to the alteration in the stress–strain curve.

A significant reduction in ultimate tensile strength and yield stress could be noticed due to the material degradation, but no significant modification of Young’s modulus in the linear portion was reported.

The only available data on the mechanical properties of PCL after UV-B exposure come from the Franca et al. study [32]. In their research, the injected PCL samples recorded an increase of 48% of the elastic modulus over the entire exposure period of up to nine weeks. The elongation at break decreased by 80% after one week of exposure and remained constant over time, while the tensile strength decreased after one week and increased after another week, then recording another decrease after four weeks of exposure followed by a strength increase up to week nine. In our study, the Young’s modulus also increased slightly after 9 h of UV-B exposure, while the UTS decreased by half for the irradiated specimens, confirming the behavior recorded by Franca et al. for the injected samples [32] and also for the 3D-printed PCL samples. This rigid and brittle mechanical behavior was attributed to the chemi-crystallization process determined by UV-B exposure [32]. 

**Figure 12:**
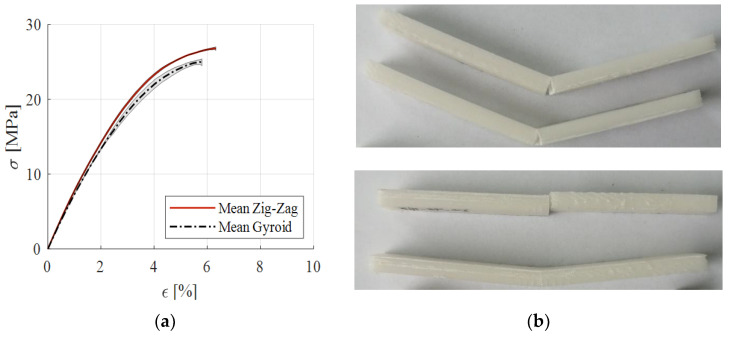
Stress–strain curves from three-point bending tests (**a**); failed flexural specimens: gyroid (**top**) and zig-zag (**bottom**) (**b**).

The mean stress–strain curves for the flexural tests are presented in Figure 12, as well as the failure patterns for the zig-zag and gyroid infills. The calculated mechanical properties are presented Table 5 as average and standard deviation (UFS—ultimate flexural strength).

As noted for the tensile testing, the gyroid printing pattern produced lower mechanical properties for all the analyzed characteristics. In our research, the results related to the flexural behavior did not confirm the producers’ data [35], with a higher value for the Young’s modulus being noted for both infill patterns.

**Figure 13:**
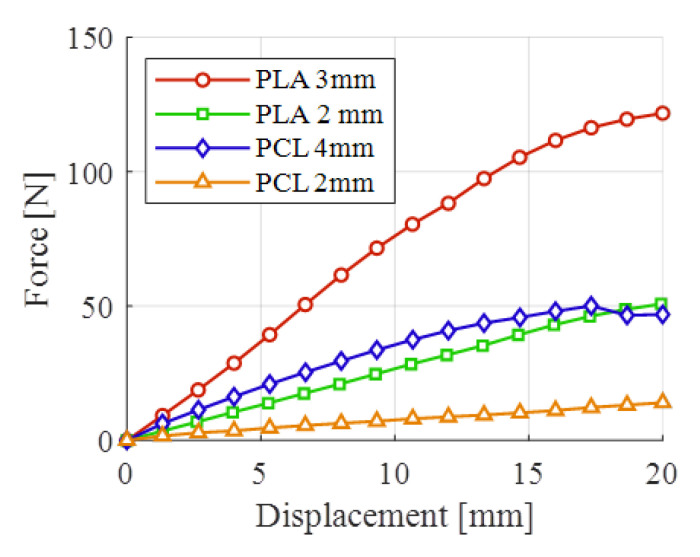
Bending tests results of semi-orthosis configurations.

The force-displacement curves for the semi-cylindrical samples are presented in Figure 13 for the analyzed material-thickness configurations. By comparing the slopes of the initial part of the curves, it can be seen that the PLA orthoses were more rigid than the PCL counterparts, with the thicker PCL configuration having a slightly higher rigidity than the thinner PLA configuration. Towards the end of the mechanical tests, the thicker PCL samples suffered a local failure, noticeable in Figure 13, through a decrease in load. The 2 mm thick PCL samples had very low stiffness, which makes this thickness unsuitable for 3DP WHOs.

### 3.5. Finite Elements Models Simulation Results

**Figure 14:**
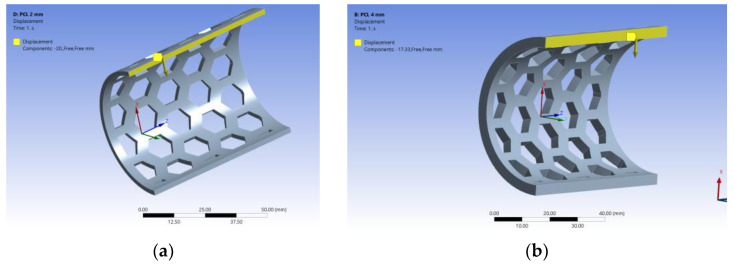
Loading the model of 2 mm thickness (**a**) and 4 mm thickness (**b**).

As a result of the mechanical tests, forces and displacements were determined for the PCL semi-cylindrical samples of different thicknesses. The obtained displacements were used in the numerical simulation to validate the models, namely to compare the forces obtained from the simulation with the experimental ones. For the 4 mm sample, a deformation of 17.30 mm was recorded (Figure 14a). This value was further used to develop the model as being the maximum force reached. For the 2 mm thick samples, the 20 mm displacement was adopted for the same reason (Figure 14b).

**Figure 15:**
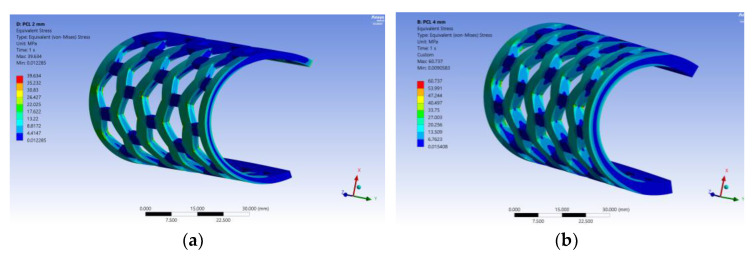
The resulting stress for the model of 2 mm thickness (**a**) and 4 mm thickness (**b**).

After running the simulations, forces of 13.509 N for the 2 mm model, and 53.143 N for the 4 mm model were obtained. Figure 15 shows the resulting stress for the semi-orthoses models, with the connection areas having the highest values.

**Figure 16:**
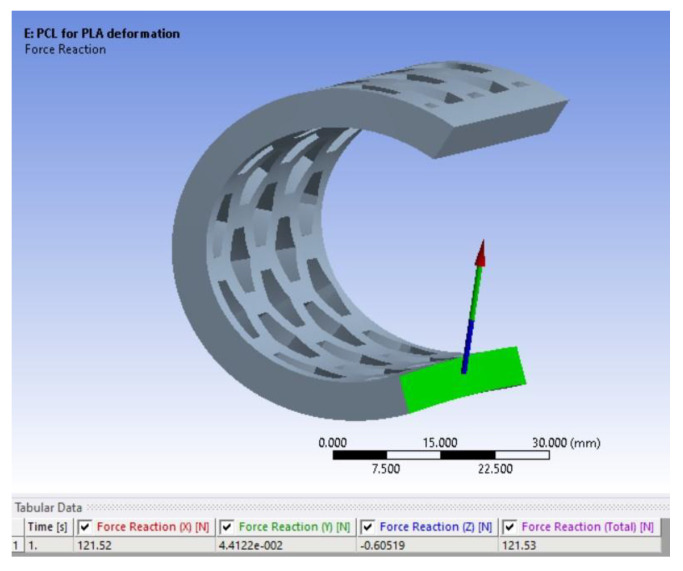
The 4.9 mm thick PCL sample providing similar stiffness as the 3 mm thick PLA sample.

Next, the model was used to identify the thickness of the PCL semi-orthosis which provides the same stiffness as the PLA semi-orthosis with a 3 mm thickness. In this sense, the objective function set in the optimization module in ANSYS was to achieve the maximum recorded force corresponding to the PLA sample of 3 mm (i.e., 121.53 N). As presented in Figure 16, this force is obtained if the thickness of the PCL semi-cylindrical samples is 4.9 mm.

## 4. Conclusions and Further Research

In this research, 3D prints made of PCL were investigated to determine their mechanical properties, the infill pattern influence over the mechanical properties, the effect of UV-B exposure on the mechanical performances, as well as their suitability in producing 3D-printed WHOs both in a vertical build orientation, and when flat and then thermoformed (flat printed orthoses required less printing time).

Calibration of the printing parameters for manufacturing good-quality samples was necessary in the absence of relevant data from the literature. Additionally, the optimal settings for the calibration parts (C-lines, calibration cubes) could not have been straightforwardly extended to taller or thicker parts with more complex designs. For instance, the 2 mm thick semi-cylindrical PCL sample showed inferior printing quality in comparison to the 4 mm thick PCL samples at the same parameter settings. However, following a time consuming calibration phase, it was possible to manufacture PCL 3DP WHO samples of good quality. On the other hand, thermoforming the flat 3D-printed PCL samples into a cylindrical shape proved unsuccessful for the tested heating temperatures and sample thicknesses. 

The mechanical characterization of the 3D-printed PCL specimens showed a good repeatability of the manufacturing process from the mechanical behavior perspective. Additionally, good dimensional accuracy and stability of the PCL parts were observed when measuring the specimens’ dimensions after the 3D printing and UV-B exposure, and comparing this data with the nominal values. The infill pattern proved not to have a significant influence over the mechanical properties. However, a relatively short time of UV-B exposure proved to have a significant negative impact on the specimens’ tensile strength.

The bending tests performed on PLA and PCL semi-cylindrical samples with open pockets showed that the 4 mm thick PCL stiffness was comparable with the 2 mm thick PLA sample stiffness, whereas a 4.9 mm thick PCL sample had a similar stiffness to the 3 mm thick PLA sample, as determined based on finite elements-based simulations of the semi-cylindrical models. 

Based on the knowledge acquired in this research, the development of antimicrobial PCL-based 3D-printed medical devices will be the main focus of future research in order to expand the variety of uses for this material beyond tissue engineering applications.

## Figures and Tables

**Table 1:** Tested parameter settings for 3D printing PCL samples.

Parameter	Bed Temperature [°C]	Printing Temperature [°C]	Printing Speed [mm/s]	Flow Rate [mm^3^/s]
Values	30; 45; 50	70; 75; 80; 85; 100; 110	10; 20; 30; 40; 50	0.9; 1; 1.1; 1.4

**Table 2:** Dimensions of specimens (nominal, 3D printed, and UV-B exposed).

Specimens	Dimensions	Nominal Dimension [mm]	Non-UV-B Specimens [mm]	9 h UV-B Specimens [mm]
1	Total length	115	114.41	114.35
	Total width	20	20.02	20.01
	Gauge width	10	10.02	10.03
	Thickness	3	3.16	3.14
2	Total length	115	114.43	114.35
	Total width	20	20.03	20.01
	Gauge width	10	10.11	10.09
	Thickness	3	3.12	3.08
3	Total length	115	114.59	114.4
	Total width	20	20.03	20.0
	Gauge width	10	10.01	10.01
	Thickness	3	3.15	3.11

**Table 3:** Mechanical properties of PCL samples.

Infill Pattern	Young Modulus (0.5%–1%) [MPa]	Poisson Ratio (0.5%–1%) [-]	Yield Stress (0.2%) [MPa]	UTS [MPa]
Zig-Zag	751.18 ± 8.7	0.29 ± 0.2	14.82 ± 0.77	18.17 ± 0.48
Gyroid	699.64 ± 14.52	0.3 ± 0.04	15.65 ± 1.16	17.61 ± 0.97

**Table 4:** Mechanical properties of UV-B irradiated PCL samples.

Specimens	Young Modulus (0.15%–0.3%) [MPa]	Poisson Ratio (0.15%–0.3%) [-]	Yield Stress (0.2%) [MPa]	UTS [MPa]
9h UV-B	767 ± 43.43	0.31 ± 0.06	7.56 ± 0.44	8.9 ± 0.45

**Table 5:** Three-dimensional-printed PCL specimens’ flexural properties.

Infill Pattern	Young Modulus (0.5%–1%) [MPa]	Yield Stress (0.2%) [MPa]	UFS (0.2 %) [MPa]
Zig-Zag	745.80 ± 8,52	17.07 ± 0.65	36.71 ± 0.2
Gyroid	698.43 ± 6.37	13.56 ± 0.5	25.09 ± 0.43

## Data Availability

Not applicable.
